# Fiber optics passive monitoring of groundwater temperature reveals three-dimensional structures in heterogeneous aquifers

**DOI:** 10.1038/s41598-024-58954-3

**Published:** 2024-04-10

**Authors:** Davide Furlanetto, Matteo Camporese, Luca Schenato, Leonardo Costa, Paolo Salandin

**Affiliations:** 1 Department of Civil, Environmental, and Architectural Engineering, University of Padova, Padova, 35131 Italy; 2 National Research Council, Research Institute for Geo-Hydrological Protection, Padova, 35127 Italy; 3 Department of Information Engineering, University of Padova, Padova, 35131 Italy

**Keywords:** Hydrology, Environmental impact

## Abstract

Alluvial aquifers often exhibit highly conductive embedded formations that can act as preferential pathways for the transport of solutes. In this context, a detailed subsurface characterization becomes crucial for an effective monitoring of groundwater quality and early detection of contaminants. However, small-scale heterogeneities are seldom detected by traditional nondestructive investigations. Heat propagation in porous media can be a relatively inexpensive tracer for groundwater flow, potentially offering valuable information in various applications. In this study, we applied passive Fiber Optics Distributed Temperature Sensing (FO-DTS) to a group of observation wells in a highly heterogeneous phreatic aquifer to uncover structures with different hydraulic conductivity, relying on their response to temperature fluctuations triggered by natural and anthropogenic forcings. A comprehensive data analysis approach, combining statistical methods and physics-based numerical modeling, allowed for a three-dimensional characterization of the subsurface at the experimental site with unprecedentedly high resolution.

## Introduction

The genesis of alluvial aquifers often induces the formation of convoluted systems of buried paleo-channels, strongly connected and hydraulically conductive, which establish preferential flow with inevitable implications on transport phenomena^1^. Consequently, identifying and correctly reproducing these preferential pathways is essential for monitoring groundwater quality and delineating the protection zone, especially in environments with a high risk of contamination^2^. To this aim, building geologically realistic models that adequately reproduce spatial connectivity is more necessary than for flow-only problems^3,4^. To address this issue, a lot of research work focused on a variety of geostatistical approaches, such as object-based and pseudo-genetic modeling^5,6^, multi-Gaussian simulations, and multiple point geostatistics^7–9^. To inform these models, prior geological knowledge remains fundamental. However, since paleo-channels and the surrounding matrix typically share the same sediment composition, nondestructive geophysical techniques often fail to detect these formations reliably^1^. In addition to intrusive measurements, such as core drilling and penetration tests, solute and thermal tracers can be used to collect information about the subsurface structure, complementing hydraulic head and flow data. Heat, in particular, has been extensively used as a tracer to investigate flow in geothermal systems, hydraulic and thermal soil properties, flow through fractured media, and exchanges between surface water and groundwater^10,11^.

**Figure 1:**
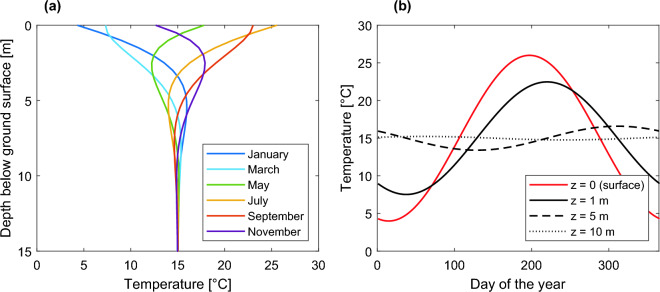
Stallman’s analytical solution: (**a**) temperature profiles along a saturated soil column forced at the top by a sinusoidal temperature boundary condition, in different months of the year. (**b**) Temperature time series at different depths. (Figure adapted from Kurylyk et al., 2015).

The temperature profile in the saturated zone of a shallow aquifer is likely to be influenced by air temperature fluctuations in its upper portion and to present seasonal oscillations dampening with depth until a point where no more influence can be observed^12,13^. In a fully saturated homogeneous soil column in contact with the atmosphere, forced by a sinusoidal temperature boundary condition on the surface, and under the hypothesis of vertical recharge and no lateral flow, the temperature profile can be derived from Stallman’s equation^12^. Stallman’s analytical solution is schematized in Fig. 1. However, this solution does not consider the possible presence of lateral flows that may be induced by the interaction of the aquifer with a river or by pumping wells. In the presence of lateral flow, significant deviations of the temperature profile can originate depending on the temperature of laterally flowing water and the magnitude of the flow. Indeed, in heterogeneous aquifers, where some layers may exhibit larger hydraulic conductivities, and consequently higher flow rates, the arrival time of the lateral thermal front is expected to be shorter for the more conductive layers, originating significant deviations in the temperature profile. Therefore, the latter can potentially be used to locate layers in the subsurface with different hydraulic conductivity. To exemplify this concept, in the Supplementary Information (SI) we illustrate a simple numerical simulation in which a lateral inflow of warmer water alters the temperature profile of a fully saturated porous medium. The corresponding results are shown in Fig. S1 of the SI.

In sufficiently permeable formations, where heat transport processes are dominated by advection, coupling the solution of the heat propagation problem with a groundwater flow model allows constraining hydraulic parameters and fluxes estimations^10,14^, either using analytical^15,16^ or numerical methods. Some early studies^17,18^ demonstrated that combining observed water levels with natural groundwater temperature fluctuations, measured by point sensors in piezometers, proved to be an effective means for estimating hydraulic conductivity through numerical model calibration. Since aquifer hydraulic properties are likely to vary much more than the rock and pore fluid thermal properties^19^, these latter do not have necessarily to be involved in the calibration procedure^10^. Considering temperature data usually results in a better estimate of hydraulic properties compared to the cases employing hydraulic data alone^11,14^.

Nevertheless, a limited number of spot temperature measurements is inherently inadequate to capture spatially variable features of the subsurface^11^. Wagner et al.^20^ and Sarris et al.^21^, for example, employed a series of downhole point sensors in thermal tracer injection tests to improve the characterization of heterogeneous alluvial aquifers by numerical model inversion. Further advances have been possible thanks to the development of Fiber Optics Distributed Temperature Sensing (FO-DTS) and its ability to provide measurements with high resolution in space and time^22,23^. Whenever the ambient water temperature is directly altered, either by heating groundwater or injecting water at a different temperature, Distributed Temperature Sensing (DTS) is referred to as “active”^22^. Conversely, “passive” DTS relies on naturally occurring temperature fluctuations, such as seasonal variations that typically occur in shallow aquifers, or those that are triggered by disturbances to the flow field^22^.

Some authors^24,25^ employed FO-DTS and circulating heated water to examine the dynamics of open boreholes in complex bedrock aquifers. The injection of water at a different temperature into the well inevitably altered the flow pattern and induced anomalies in the temperature-depth profile that allowed the identification of hydraulically active fractures. Alternatively, Banks et al.^26^ proposed using FO-DTS assisted by electrical heating to detect active fractures under ambient groundwater flow conditions. Electrical Joule heating was provided by electrical cables installed alongside the fiber optics and the technique allowed the authors to track the ambient groundwater flow conditions without perturbing the flow pattern. More recently, Pouladi et al.^16^, proposed an analytical framework whereby passive DTS data can be used to dynamically quantify the vertical flow profile along a borehole in a fractured aquifer. The approach was validated by means of a numerical model and successfully employed in a field experiment.

In wells, conduction through the surrounding rock and horizontal advective transport are the main driving factors of changes in the temperature profile when vertical water flow is minimal. Significant temperature gradients can induce free-convection (buoyancy) effects altering the observed temperature field. However, Eppelbaum and Kutasov^27^ estimated that free-convection contributes only up to 0.05 °C in boreholes deeper than 20 m and is minimized in wells with small diameters. Therefore, the free-convection of slightly heated water with minimal mixing has a negligible impact on temperature dynamics, resulting in temperature variations that are well below the accuracy of FO-DTS systems.

Within the context of granular media, Klepikova et al.^28^ employed downhole DTS to monitor a coupled solute and heat tracer test in a layered alluvial aquifer. In this framework, high-resolution temperature measurements allowed the authors to invert the saturated hydraulic conductivity field of a numerical model, considering small-scale heterogeneities by means of a pilot points parameterization^29^. The combination of tracer test DTS data with a sufficiently flexible calibration technique proved to be a valuable tool for aquifer characterization at a high level of detail.

Overall, most temperature-supported applications in alluvial aquifers were conducted using active experiments, with consequent limitations on the temporal and spatial scales of investigation^20,21,28,30^. Differently from non-reactive solutes that are commonly used in tracer tests, heat has a nonconservative nature, which limits the distance at which significant distinct temperature anomalies can be induced^31^. Moreover, due to a thermal retardation effect^32^, covering the same area of investigation would require the application of the thermal tracer for a longer duration compared to solute tracers^31^. These factors, naturally, have consequences on the costs of the experiments (e.g., the power supply of the heating devices). Therefore, passive temperature sensing may be more suitable for long-term and large-scale monitoring.

To the best of our knowledge, downhole DTS was never employed in passive mode to detect paleo-channels in alluvial aquifers. The main objective of this work is to explore the use of passive FO-DTS as an alternative to active thermal tracer tests, to inform the estimation of the heterogeneous hydraulic conductivity field in an alluvial phreatic aquifer embedding hydraulically conductive paleo-channels. High-resolution temperature transient profiles, collected in a group of seven piezometers at the experimental site, were analyzed and employed to calibrate a three-dimensional (3D) model via pilot points.

## Methods

### The study area

**Figure 2:**
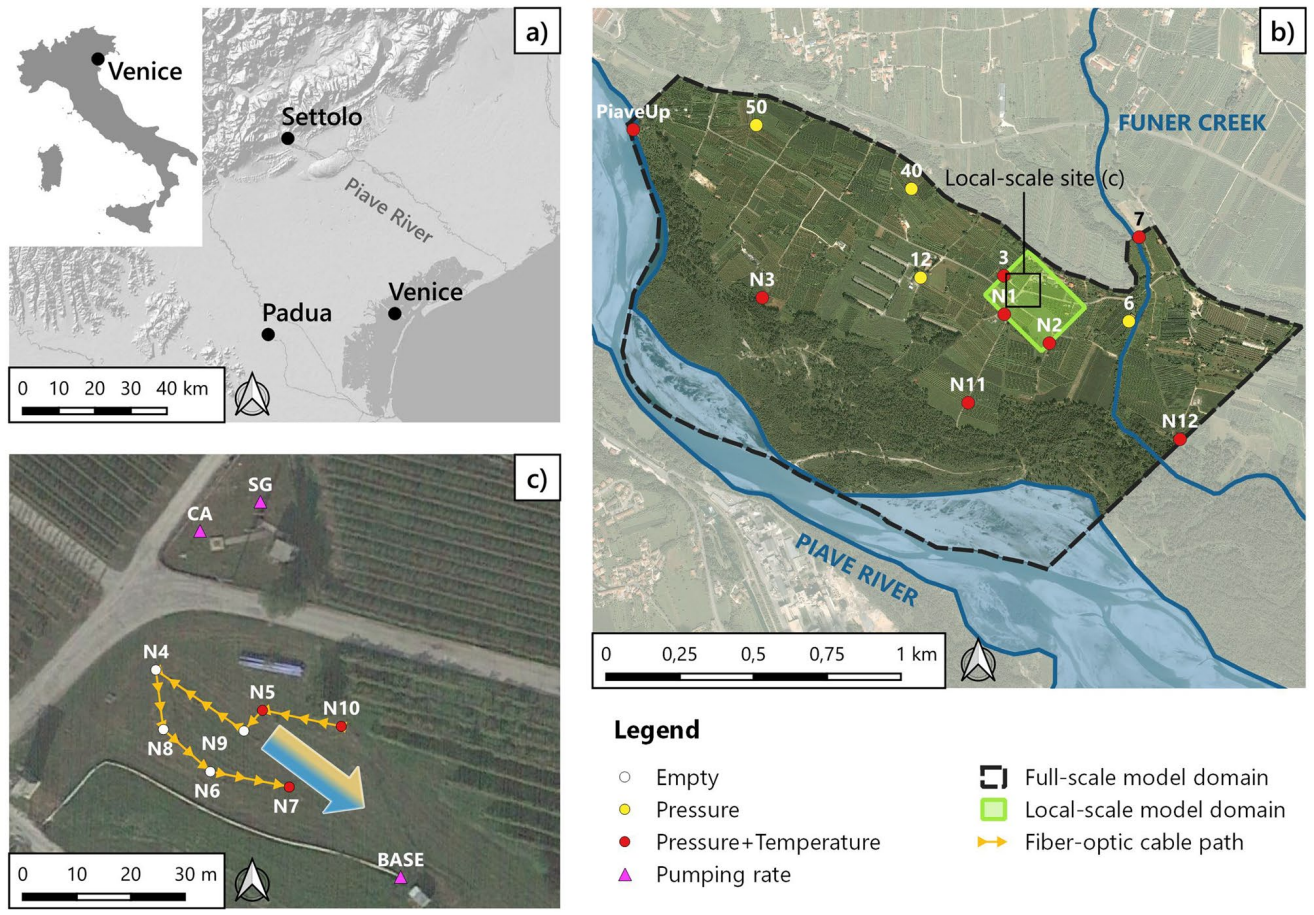
Geographic framework: (**a**) location of the Settolo study area within Italy and the Veneto Region. (**b**) Overview of the full-scale experimental site, with the position of the observation wells; colors are related to the variables measured by the point probes, as indicated in the legend. The image also shows the domain of the local-scale model, highlighted in green. (**c**) Local-scale study area with the position of the pumping wells and of the fiber-optic cable path in the group of investigated boreholes. The direction of the large arrow indicates the mean groundwater flow direction, while the filling color represents the groundwater temperature gradient observed in the data. (Figure created with QGIS v.3.28.4-Firenze, https://qgis.org/en/site/).

The experimental site of Settolo is located within the municipality of Valdobbiadene, in the north-eastern Italian province of Treviso, about 60 km from Venice and Padova (Fig. 2a). The site lies at the foot of the Venetian Prealps, where the Piave River generated a sedimentary sequence of alluvial deposits that allowed the formation of a shallow unconfined aquifer, whose thickness ranges from about 30 to 60 m, overlying a conglomerate formation that acts as an impermeable bedrock^33^. The aquifer is exploited for irrigation and drinking water supply and has been extensively studied for more than ten years^4,8,33–36^.

**Figure 3:**
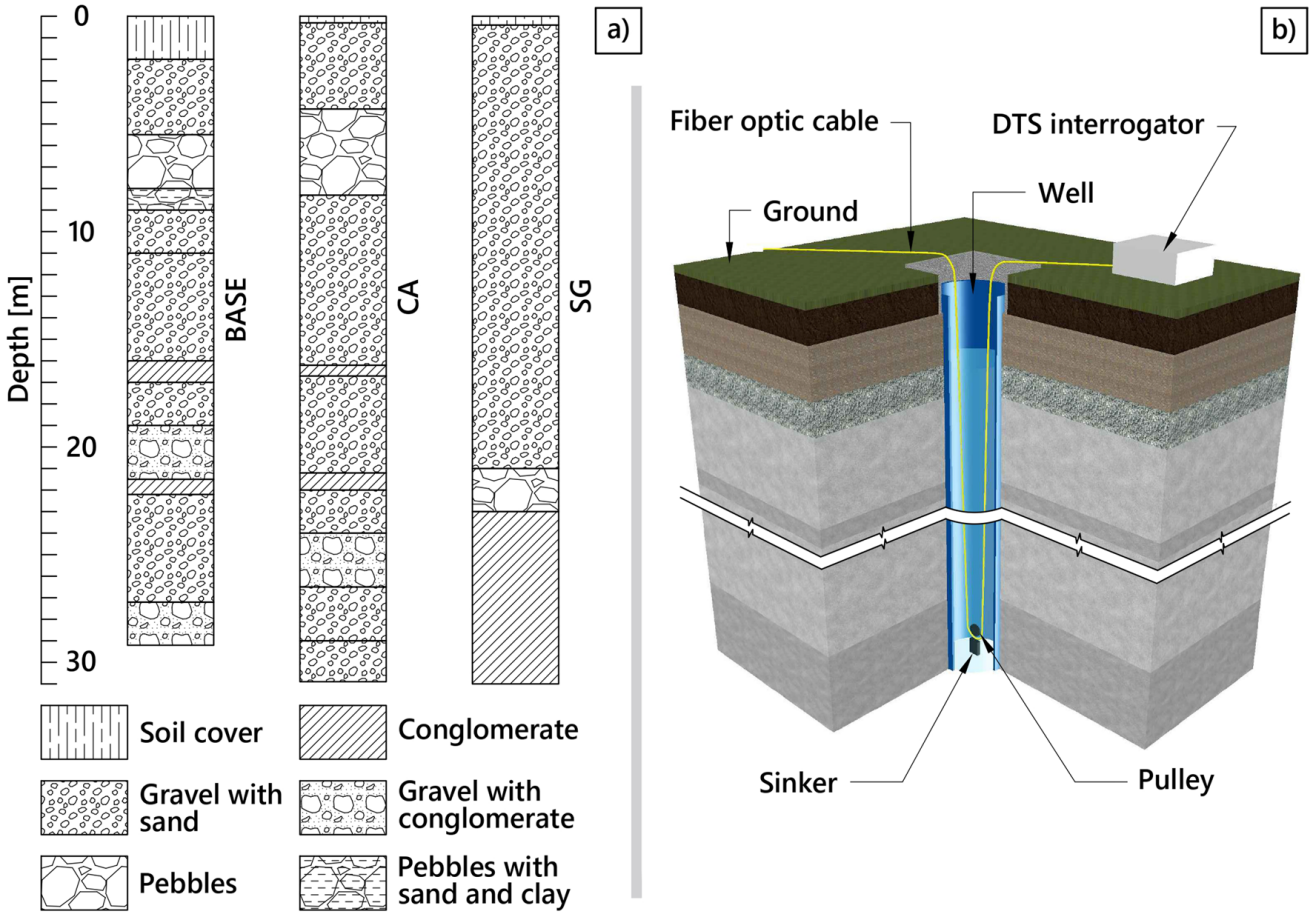
(**a**) Stratigraphic profiles obtained during drilling of the three pumping wells BASE, CA, SG (Fig. 2c), with indication of the lithofacies. (**b**) A schematic of the installation of the fiber optic cable within a borehole. (Figure created with AutoCAD 2024, https://www.autodesk.it/).

The main source of groundwater recharge is the Piave River, with a limited contribution from the Funer Creek (Fig. 2b). Empirical evidence emerged about the presence of a network of highly hydraulically conductive paleo-riverbeds, interspersed within a matrix of sandy-gravelly soils^4,8^, which may establish preferential paths in the flow field. Figure 3a shows the stratigraphic profiles obtained in the past from the drilling of the three pumping wells BASE, SG, and CA (Fig. 2c). Although these three stratigraphies cannot be considered as fully representative of the area due to its intrinsic high variability, they provide a general tendency of the expected layering of geomaterials in the site. Considering the importance of this aquifer for its extensive use and the concurrent agricultural destination of the overlying soil, with the application of fertilizers and pesticides^35^, improving the knowledge on the hydraulic properties and the spatial distribution of the aforementioned preferential paths becomes crucial for effectively monitoring the groundwater quality.

Nested within a full-scale monitoring network (Fig. 2b), a local-scale system of observation wells allows for the conduction of experiments over an area of about 0.3 ha (Fig. 2c). In particular, point sensors Beaver ATP 10 (AquiTronic) and DL/N 70 (STS) continuously measure the water table depth at the observation wells indicated in yellow in Fig. 2b,c, as well as single-point water temperature at the observation wells indicated in red, both with a temporal resolution of 10 min. The three pumping wells managed by the local water utility company for drinking water supply, shown in Fig. 2c, are equipped with flowmeters measuring the pumping rate. In particular, the BASE pumping well can extract up to 180 l/s, representing the most significant contribution if compared to the maximum rates of 27 l/s and 22 l/s of CA and SG, respectively.

### Experimental setup and data collection

Between September 18th, 2020 and October 14th, 2020 a FO-DTS campaign was carried out in the local-scale study area to acquire vertical transient water temperature profiles along the depth of the piezometers. The optical fiber cable employed in this installation is a commercial cable, armored with stainless steel loose tube hosting a couple of 50/125 G.651 polyimide coated fibers, surrounded by stainless steel strands, up to an overall diameter of approximately 3 mm. The cable is specifically designed for a fast thermal response. One single cable of sufficient length to connect each borehole was installed at the site, tracing the path depicted in Fig. 2c. The cable was deployed in a loop configuration within every borehole and appropriately anchored to protect and stabilize it while it bends at the bottom. The depth of the boreholes is approximately 30 m and their diameter measures 0.10 m.

A commercial DTS interrogator with a spatial resolution of 1 m was used to probe the cable in double-end configuration^37,38^. The DTS system employed here is an optical time domain reflectometer (OTDR) system from LIOS sensing. A typical Raman OTDR scheme can be found in Schenato (2017). The acquisition time resolution was 10 min, corresponding to expected repeatability within a few tenths of °C, and the accuracy within a 30 km range resulted within $\pm 1$ °C. A schematic of the downhole installation of the cable is shown in Fig. 3b.

During the experimental campaign, the pumping wells were repeatedly activated and deactivated to meet the drinking water demand, and the water utility company provided the detailed pumping rate time series. Precipitation and other weather data needed to compute the reference evapotranspiration were collected from the Regional Environmental Protection Agency (ARPAV) meteorological stations of Bigolino, about 3 km south-east of the site, and Quero Vas, about 5 km north-west. The position of the water table and the groundwater point temperature, measured by sensors of the monitoring network described in the previous section, were also available in the piezometers indicated in red in Fig. 2b,c, with the exclusion of PiaveUp, N03, and p07, for 14 months ahead of the experiment. However, the water level variations and temperature of the Piave River are well correlated by those measured in N12^35^. In the local-scale area, in particular, discrete temperature data are available in N10, N05 and N07 (Fig. 2c) with an accuracy of $\pm 0.1$ °C, from probes located about 5 m below the water table. However, point temperature measurements in N07 were discarded due to anomalous data.

### Data analysis and numerical modeling

#### Data analysis

The lowest position of the water table reached during the experimental campaign was approximately 5 m below the ground level. A preliminary inspection of the data showed that, above the water table, in-well temperature dynamics were dominated by air temperature fluctuations, propagating in the dry upper part of the piezometers. For the objectives of this study, however, data analysis and numerical modeling focused on the saturated portion of the subsurface, where the fiber-optic cable measured the actual groundwater temperature. The raw temperature data measured by the DTS showed an offset bias, which was corrected by a constant and homogeneous shift of −0.78 ^∘^ C, obtained by comparing the signal of the discrete traditional temperature probe in N10 and N05 with the DTS time series collected at the corresponding depths. Additionally, a moving average of 5.5 hours window was applied to the DTS data to maximize the correlation with the aforementioned discrete temperature probe signals. A comparison between the raw DTS data, the corrected DTS data, and the point probes signals in N05 and N10 is shown in Fig. S9 of the SI.

Pearson correlation coefficients between time series at each depth were computed to highlight similarities and dissimilarities of the signal dynamics both within the same well and between different boreholes. Temperature time series collected by the DTS along the depth of the investigated boreholes present peculiar short-scale fluctuations, mainly related to the pumping activity. The objective of analyzing the correlation of temperature signals collected at different depths is to highlight those depths presenting similar responses (not similar temperatures), so as to identify the position of “layers”, intended as portions of the profile, whose dynamic behavior is similar. A cluster analysis of the temperature time series collected at different depths was also performed for each piezometer to identify possible layers exhibiting analogous responses to water table variations, induced mainly by the Piave River and water withdrawals in the nearby pumping wells.

#### 1D numerical modeling

Temperature observations were preliminarily compared with the solution of a one-dimensional (1D) vertical finite element model simulating purely conductive heat propagation in a homogeneous water column. The 1D model aims at reproducing the temperature field that, given the initial temperature profile and the bottom and top boundary conditions, would evolve in time without considering lateral thermal fluxes in the piezometer. Therefore, it simply resolves for the one-dimensional heat equation in a homogeneous medium:

1 $$\begin{aligned} \frac{\partial T}{\partial t}= \frac{\lambda _{l}}{\rho _{l} c_{l}} \frac{\partial ^2 T}{\partial z^2} \end{aligned}$$

where *T* (K) is the temperature, *t* (s) is time, *z* (m) is the spatial (vertical) coordinate, $\lambda _{l}$ (W m^-1^ K^-1^) is the thermal conductivity of water and $\rho _{l} c_{l}$ (MJ m^-3^ K^-1^) is the volumetric heat capacity of water. A 26-m-high 1D domain was modeled for all piezometers, simulating the water column in the saturated zone. The transient boundary conditions assigned on the top and at the bottom of the simulated water column consist of imposed temperature (Dirichlet or first-type boundary conditions), with values taken from the data collected by the FO-DTS at the corresponding depths. The measured temperature profile at the beginning of the experiment was imposed as initial condition. The model parameters (water thermal conductivity and heat capacity) are reported in Table 1. Appreciable vertical components of the hydraulic gradient may temporarily appear in the proximity of the water table during the activation/deactivation of the pumping well, but they rapidly dissipate as the new dynamic equilibrium condition of the water table is reached. These vertical gradients could potentially perturb the temperature field in the piezometer. However, considering that the maximum measured excursion of the water table related to pumping is approximately 0.4 m, while the spatial resolution of the FO-DTS is 1 m, and considering that the accuracy of the DTS measurements in this study is within ±1 C^∘^, we can reasonably assume that these perturbations did not affect significantly the measured temperature profiles. Under this assumption, the temperature anomalies obtained by subtracting temperatures simulated by the 1D model from the data might indicate the contribution of the horizontal heat fluxes, mainly governed by advection, to the measured temperature profiles, similar to what suggested by Saar^14^.

**Table 1:** Fixed (i.e., not calibrated) model (both 1D and 3D) parameters and their values.

Parameter	Symbol	Value	Unit
Specific yield	$S_y$	0.3	–
Specific storage	$S_s$	$10^{-4}$	m^-1^
Porosity	*n*	0.4	–
Thermal fluid-expansion coefficient	$\beta$	$1.6\cdot 10^{-4}$	K^-1^
Thermal conductivity of solid phase	$\lambda _{s}$	3.0	W m^-1^ K^-1^
Thermal conductivity of water phase	$\lambda _{l}$	0.65	W m^-1^ K^-1^
Volumetric heat capacity solid phase	$\rho _{s} c_{s}$	2.52	MJ m^-3^ K^-1^
Volumetric heat capacity of water phase	$\rho _{l} c_{l}$	4.2	MJ m^-3^ K^-1^
Longitudinal thermal dispersivity	$\alpha _L$	5.0	m
Transverse thermal dispersivity	$\alpha _T$	0.5	m

#### 3D numerical modeling

A 3D finite element model of the local-scale area was implemented using FEFLOW 7.5^39^ (DHI), coupling the transient solution of the groundwater flow equation and the density-dependent heat transport equation in porous media under the hypothesis of the Oberbeck-Boussinesq approximation^39^. The mesh has a rectangular base, highlighted in green in Fig. 2b, covering approximately 0.6 km^2^. It is refined horizontally around the FO-DTS experiment boreholes and has 32 vertical layers of 1 m each, with nodes corresponding to the spatial resolution of the DTS observations. The simulation period extends from August 18th, 2020 to October 14th, 2020, starting one month in advance with respect to the DTS experiment period, to reduce the effects of the initial conditions. Hydraulic head and temperature boundary conditions for the north-west (NW) and south-east (SE) sides of the domain were obtained from a large-scale finite element model of the entire Settolo aquifer, whose boundaries are depicted by the black dashed line in Fig. 2b. Further details on this latter model can be found in the SI (Fig. S2–S6). The same model showed that the principal flow direction in the local-scale area is from NW towards SE so that a no-flow and no-thermal flow boundary condition could be imposed on the north-east (NE) and south-west (SW) sides of the local-scale domain.

Similarly, no flow or heat exchanges were considered to occur through the bottom of the domain. Daily precipitation and reference evapotranspiration, computed using the FAO Penmann-Monteith equation^40^, along with daily-averaged air temperature, were used as boundary conditions on the surface. The hourly-aggregated pumping rates at the nodes corresponding to the screened interval of the pumping wells BASE, CA, and SG (Fig. 2c) were imposed as sink terms.

The model calibration demonstrated that the main parameter controlling the propagation of the thermal plume in the 3D local-scale model is the saturated hydraulic conductivity, while subsequent changes to the thermal dispersion coefficients and the porosity yielded no significant improvements. Thus, their default values were not modified for the following forward simulations. These are reported in Table 1, along with the values of other model parameters not subjected to calibration.

The saturated hydraulic conductivity field was calibrated using FePEST, the FEFLOW-integrated extension for PEST^41^ (Model-Independent Parameter Estimation and Uncertainty Analysis). The procedure aimed to recreate a hydraulic conductivity field that could explain the pattern of temperature responses revealed by the FO-DTS campaign. The software minimizes a weighted sum of squared residuals objective function using the Gauss-Levenberg-Marquardt Algorithm (GLMA)^41^. Hourly resampled temperature signals measured by the DTS between 5 m and 30 m below the ground, corrected as described above, were used as observations, together with the measured hydraulic heads in N05 and N10. A weighting factor of 25 was assigned to each of the latter to make them comparable with an entire temperature profile. Model parameterization was performed using pilot points distributed over ten equally spaced layers of 92 pilot points each, more densely located around the observations. 3D Ordinary Kriging was employed as an estimator of the continuous conductivity field, setting an anisotropic exponential variogram with ranges of 48 m in the longitudinal horizontal direction (corresponding to the principal flow direction), 10 m in the transverse horizontal direction and 5 m in the vertical direction. The ratio between horizontal and vertical hydraulic conductivity was set to 10.

## Results

### FO-DTS data

**Figure 4:**
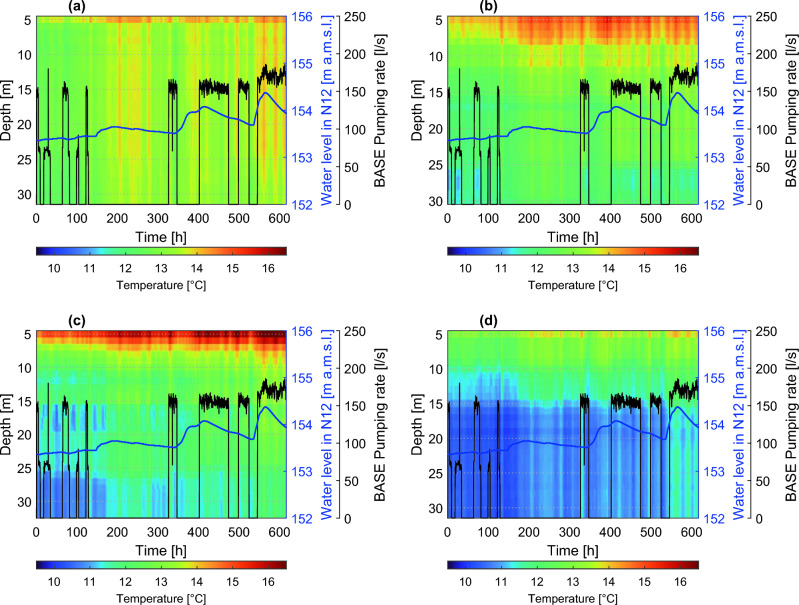
Temperature data measured by the FO-DTS as a function of time and depth in boreholes N10 (**a**), N05 (**b**), N09 (**c**), and N07 (**d**). Time is indicated in hours from the beginning of the experiment. Color bars indicate the temperature in °C. Water level measured in borehole N12 in m above the mean sea level, along with BASE well pumping rate in l/s, are also reported. The water level in N12 is shown as it is representative of the piezometric surface dynamics, mainly controlled by the Piave River and undisturbed by the pumping process.

Figure 4 shows color maps of temperature data collected during the experiment in boreholes N10, N05, N09, and N07 as a function of time and depth, along with the pumping rate in the BASE well and the water level recorded in N12 (representative of the Piave River). These four boreholes (out of the seven included in the survey) were chosen as they represent the different temperature dynamics observed in the field.

The mean flow direction in the experimental area goes from NW towards SE, as represented by the direction of the arrow in Fig. 2c. The temperature signals collected by the FO-DTS, averaged in time and along the depth of each borehole, reveal a spatial temperature gradient that is transversal with respect to this mean flow direction, with groundwater temperature decreasing from NE towards SW, as indicated by the filling color of the arrow in Fig. 2. Note, for example, the average higher temperatures in N10 compared to N07 (Fig. 4). Additional explanations and figures related to this point are reported in the SI (Figs. S7 and S8). At the same time, the temperature recorded by the fiber optics at each depth tends to increase in time within the acquisition window, indicating an advancing warmer thermal front (in the direction of the mean groundwater flow). This is consistent with the seasonality of groundwater temperature related to the Piave River water temperature fluctuations propagating into the aquifer and can be observed, for example, in the point-temperature signal of well p03 (Fig. S5 of the SI), which can be considered an upgradient control point for the local-scale domain.

The FO-DTS data revealed that the response of the temperature field to external forcings, mainly the warmer water coming from the Piave River and the pumping well activation/deactivation sequence, is not homogeneous. Our interpretation of this experimental evidence is that significant heterogeneities in the thermal response, especially at such a small spatial scale, can be possibly attributed to heterogeneities in the distribution of the soil properties, with advective heat transport likely occurring with a faster dynamics within the more conductive layers. In particular, wells N05 and N09 display a response that differs markedly in some depth ranges, presumably due to the presence of layers with different hydrogeological properties.

Observing borehole N05 in Fig. 4, temperature abruptly decreases in the depth range between 26 m and 30 m in response to the activation of the BASE pumping well, returning to higher values, more homogeneous along the vertical, when pumping is turned off, the flow field returning to its ambient conditions. This evidence may suggest the existence of a soil layer with higher hydraulic conductivity that connects the BASE well with colder regions of the aquifer, thus explaining the rapid temperature decrease in response to pumping activation driven by advective mechanisms. The N05 piezometer also shows a thin layer at 17 m with a lower average temperature, perhaps due to lower conductivity that retards the warmer thermal front. The N09 well exhibits a rather complex vertical heterogeneous response to external forcings, probably due to the presence of a sequence of layers with different hydraulic conductivity. Conversely, piezometer N10 displays a vertically homogeneous behavior, with a uniform average temperature profile, generally warmer and unaffected by the pumping events. Borehole N07 displays yet another behavior along its depth, with the top 10–15 m thick layer characterized by warmer temperatures and generally colder layers from 16 m to 30 m depth, as can be noted in Fig. 4.

**Figure 5:**
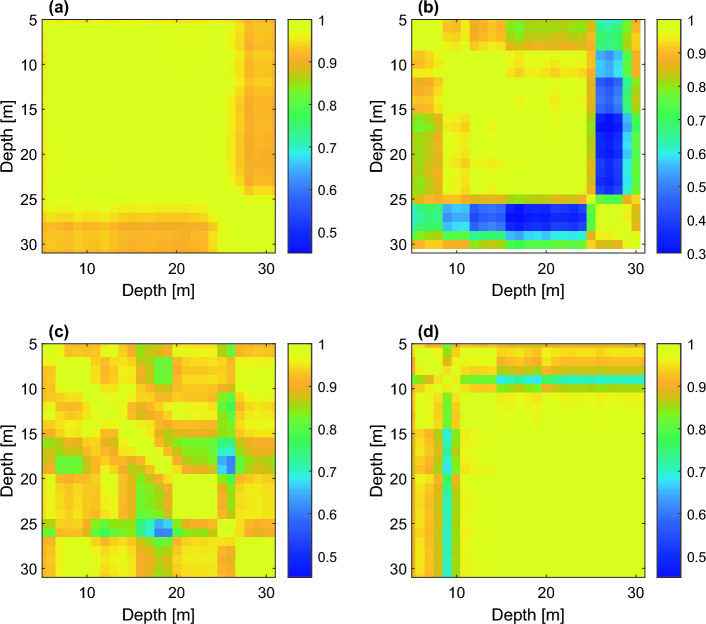
Color maps of the Pearson correlation coefficients between time series recorded at different depths, in meters, within the four representative boreholes (**a**–**d**). Note that the color scale is different for N05 (**b**).

Figure 5 shows color maps of Pearson correlation matrices between temperature time series collected at different depths in the same well, for the four representative boreholes. The correlation plot of N05 shows a significantly lower correlation between temperature time series in the depth band 26-30 m and the upper 5–25 m due to the different response of a presumably more conductive layer below 26 m to the BASE pump activation, already highlighted in the visual inspection of the data. Regarding N09, the substantial heterogeneity of its correlation plot suggests the presence of alternate layers exhibiting different dynamics. In contrast, the correlation map for N10 confirms a substantial similarity of the temperature signals recorded throughout the profile, with the absence of layer-dependent behaviors in response to external forcings, indicating a homogeneous soil texture profile. For N07, the soil layer around 9 m depth is poorly correlated with all the other layers. Upon close examination of Fig. 4, this could be attributed to more pronounced variations of temperature at this depth in response to pumping activity. A complete Pearson correlation matrix is reported in Fig. S10 of the SI, while the dendrograms resulting from the cluster analysis are shown in Fig. S11, confirming the existence of heterogeneous soil layers in boreholes N05 and N09.

### Simplified 1D heat model

**Figure 6:**
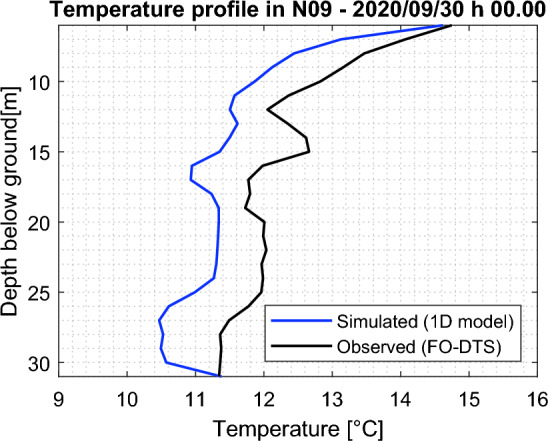
Comparison between a single temperature profile simulated by the 1D model and the corresponding experimental data, in well N09.

**Figure 7:**
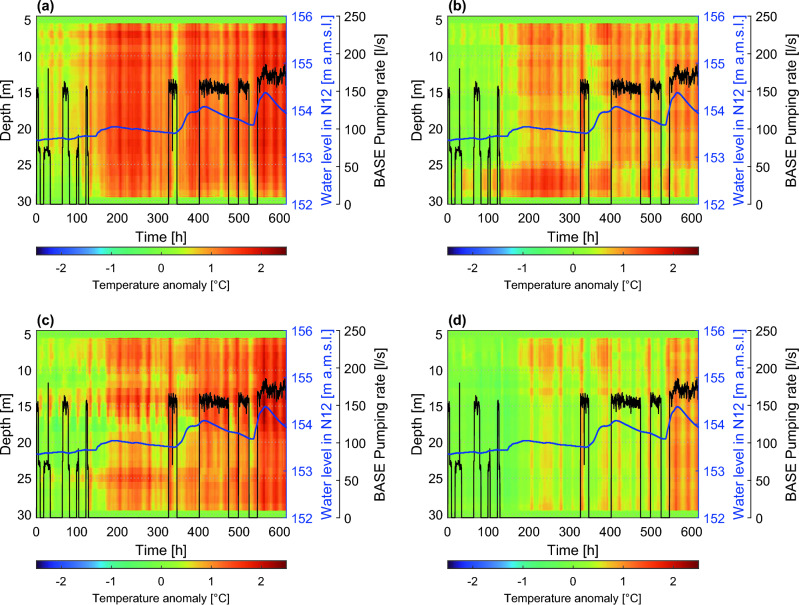
Temperature anomalies of FO-DTS data with respect to the solution of the 1D heat transfer equation in a homogeneous water column, as a function of time and depth in boreholes N10 (**a**), N05 (**b**), N09 (**c**), and N07 (**d**). Color bars indicate temperature anomalies in °C. Water level in N12 and BASE well pumping rate are also reported.

A comparison between a single profile obtained by the 1D model (for well N09) and the experimental data, at a given time instant, is shown in Fig. 6. A complete overview of the results of the 1D model is reported in the SI (Fig. S12). The anomalies obtained by subtracting the temperatures simulated by the 1D model from the FO-DTS data are shown in Fig. 7. Considering the advancing warmer thermal front observed in the data and the purely conductive nature of this model, positive temperature anomalies hint at horizontal advective contributions of warm water propagating within the aquifer. In wells N05 and N09, the warmer thermal front appears to be non-homogeneous along the vertical, suggesting possible heterogeneities in the aquifer properties. A few slightly negative temperature anomalies in some layers hint at a horizontal inflow of cold water, suggesting a delay in the arrival time of the advancing warm water thermal front, which could be attributed to a lower hydraulic conductivity of those layers. Furthermore, these advective contributions appear to be mainly correlated with the water level observed in N12, reported in Fig. 7 with a blue line, which is assumed to be representative of piezometric surface fluctuations in the domain, controlled by the water level of the Piave River.

The temperature anomaly for N05 highlights the same distinct layer previously identified at the bottom of the piezometer. Figure 7 suggests for N09 the existence of three main hydrogeological structures with higher hydraulic conductivity, between 25 and 30 m (possibly contiguous to the one of N05 if we also consider their spatial proximity), in the depth range of 14–17 m and approximately above 10 m depth. The temperature anomaly in N10 shows a clear convective contribution to its temperature profile, compared to the other wells, without exhibiting significant vertical differentiation. These findings can be explained by hypothesizing a stronger connection to the Piave River, as if N10 was enclosed in a hydraulically conductive and vertically homogeneous soil formation where the advancing thermal front moved faster. Regarding N07, in contrast, the corresponding comparison with the purely conductive 1D heat transport model suggests a poor advective transverse contribution to the temperature profile, which may indicate that well N07 lies within a region of the domain having lower hydraulic conductivity, showing little heterogeneities along the vertical.

### Complete 3D heat transport model

**Figure 8:**
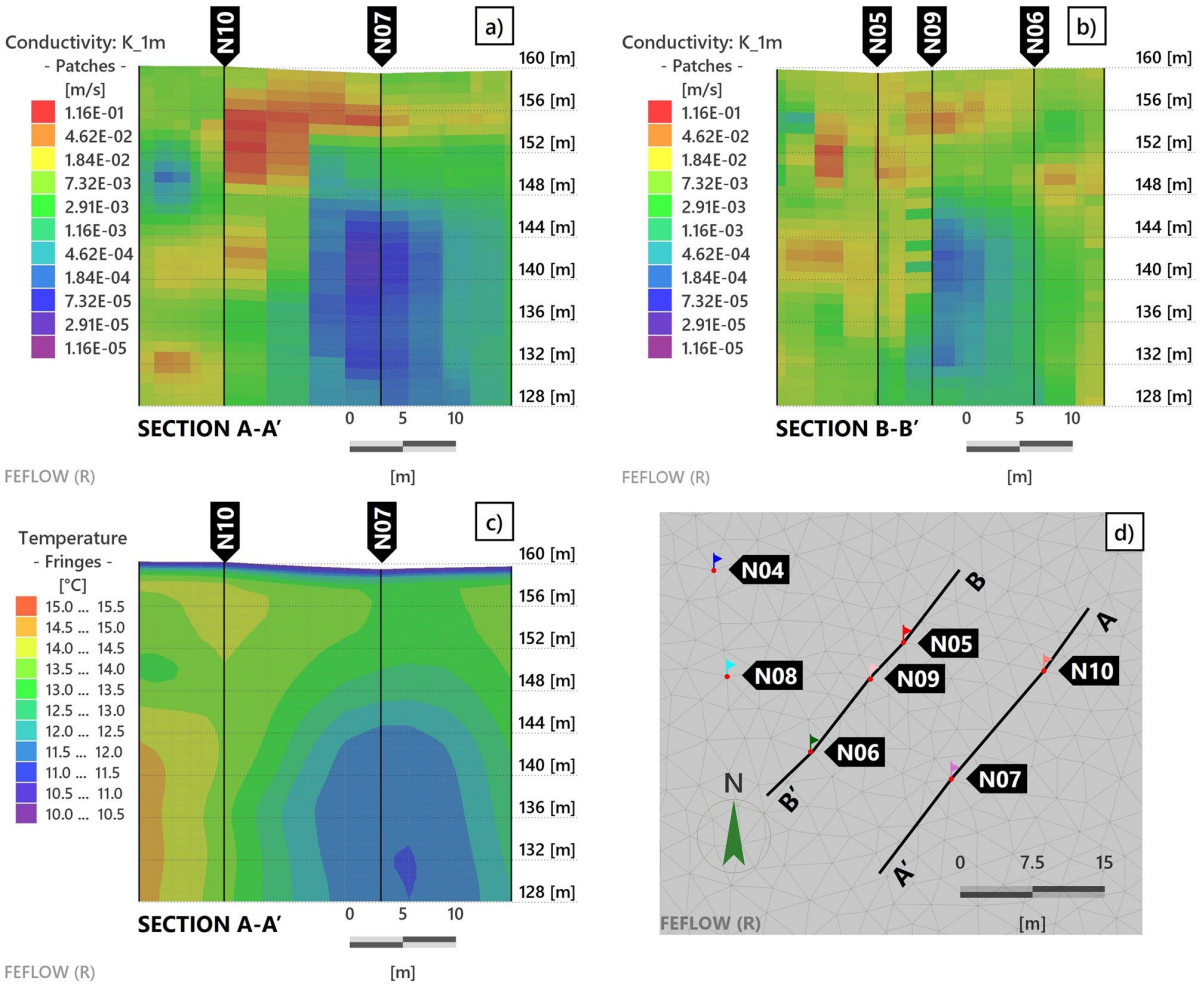
Images (**a**–**b**) show the saturated hydraulic conductivity field resulting from model calibration in sections A-A’ and B-B’, whose position is indicated in image (**d**). Note that the color scale of the hydraulic conductivity is logarithmic and that elevations are reported in meters above the mean sea level. Image (**c**) shows the simulated temperature field in section A-A’ at the final simulation time step. (Figure created with FEFLOW v. 7.5, https://www.mikepoweredbydhi.com/products/feflow).

Figures 8a and b show the saturated hydraulic conductivity field resulting from the automatic calibration of the 3D model along the two sections indicated in Fig. 8d. The inversion procedure pushed toward the emergence of strong heterogeneities, with hydraulic conductivities varying by up to three orders of magnitude along the same borehole. However, it can not reproduce the characteristic vertical layering, where relevant, shown by the experimental data and previously illustrated. The estimated horizontal hydraulic conductivity values range from about $10^{-1}$ m/s to $10^{-5}$ m/s.

Nonetheless, it can be observed that the calibrated hydraulic conductivity field displays higher values, more vertically homogeneous, in correspondence with N10 and N05. At the same time, a wider variability range appears along the vertical of N07, with very low values below 10 m of depth (149 m a.m.s.l.). An intermediate behavior arises for N09, with significant heterogeneities. These findings are in substantial agreement with the results of the previous analysis. Figure 8c displays the simulated temperature field in the final time step of the simulation, which highlights a strongly homogeneous temperature profile along the N10 vertical, while on the N07 vertical the temperature decreases to a depth of 15 m (144 m a.m.s.l.) and is more homogeneous below, following the experimental data. Moreover, the model can reproduce the transverse temperature gradient (from left to right in Fig. 8c) observed in the data.

**Figure 9:**
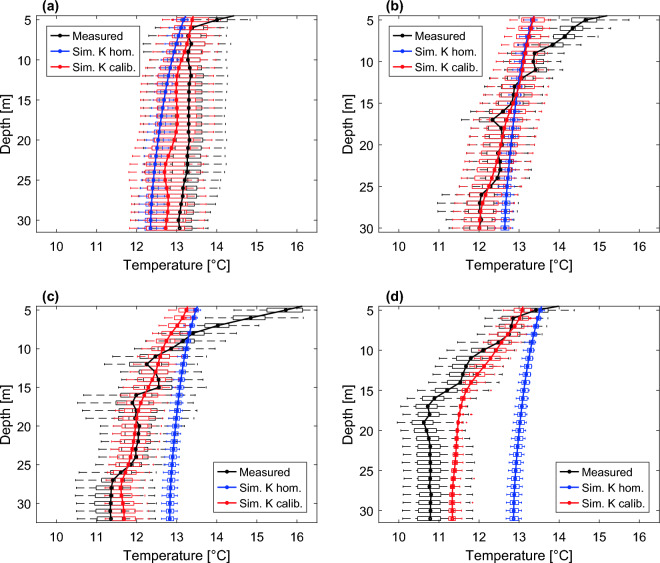
Box plots and mean profiles of DTS experimental data (black), simulated temperatures with homogeneous hydraulic conductivity field (blue) and heterogeneous conductivity field resulting from model calibration (red), along boreholes **a**) N10, **b**) N05, **c**) N09, and **d**) N07.

Time-averaged profiles and box plots of the simulated temperature time series at different depths, before and after model calibration, are shown in Fig. 9, together with those of the corrected experimental data. It is clear that the calibration, which resulted in the heterogeneous hydraulic conductivity field described above, significantly improved the simulated temperature profiles compared to the same model with a homogeneous field. However, the simulated profiles in Fig. 9 also confirm the inability of the calibrated model to accurately reproduce the layer-dependent temperature signatures that characterize the data.

## Discussion

A FO-DTS survey was conducted to collect transient temperature profiles along the depth of a local-scale group of piezometers with a spatial resolution of 1 m. In contrast to active DTS, where water is artificially heated to enhance the detection of temperature variations, the technology used in this study was passive DTS, representing a lower-cost alternative that employs naturally occurring and flow-triggered temperature fluctuations. Klepikova et al.^42^ showed that passive temperature tomography, although not performed with fiber optics, was sufficient to identify preferential pathways in a fractured aquifer under the combination of different pumping conditions. Furthermore, passive DTS requires less practical and technological effort, making it more suitable for applications over large spatial scales and for long-term monitoring^43^.

In the case study presented here, the experimental data provided notable insight into the spatial arrangement of the heterogeneous structures characterizing the investigated aquifer, which could hardly be inferred with a similar level of detail using other borehole techniques. This exemplifies how FO-DTS can be a transformative technology^23^ with the potential to enhance our ability to understand hydrological processes^44^, thanks to its inherent sampling modality through consecutive discrete intervals. However, the complexity of the site was inevitably reflected in the observed temperature field, making the interpretation of the data challenging. For this reason, we relied on the combination of different approaches, from statistical data analysis to 1D and 3D heat transport modeling.

Indeed, fiber-optic temperature data collected during this kind of experiments are suitable as observations in the calibration of three-dimensional numerical models, as they can provide much more information than the traditional point-wise temperature observations, which do not permit reaching the same resolution. For instance, Klepikova et al.^28^ successfully estimated the hydraulic conductivity field in an alluvial aquifer using pilot point parameterization and DTS data from heat tracer tests, obtaining a good fit between the simulated and observed variables. In our case study, the measurements showed a transversal temperature gradient with respect to the principal groundwater flow direction, which required providing heterogeneous boundary conditions on the NW side of the local-scale model to be reproduced. These boundary conditions were obtained from a full-scale aquifer model, which presented a significant level of uncertainty since it was calibrated based on point temperature measurements and reconstructed boundary conditions.

To further address this limitation, we defined a local-scale model domain in which the area of interest was sufficiently apart from the boundaries. On the other hand, increasing the mesh size requires many pilot points to adequately cover the domain, leading to an inevitable increment of the computational burden.

Despite these limitations, the hydraulic conductivity field resulting from the calibration confirmed the overall hydrogeological features that emerged from the data analysis and from previous knowledge. Naturally, in the absence of specific field investigations, particularly with such a level of detail, it is not possible to quantitatively compare the estimated hydraulic conductivity field with real-world data. Nevertheless, a rough qualitative comparison can be made with the available stratigraphic profiles. We can observe (Fig. 3) that the main geomaterial is gravel with sand, interspersed with layers of coarser gravel or pebbles between 5 m and 10 m below the ground surface (larger hydraulic conductivity) and layers of conglomerates (lower hydraulic conductivity) appearing and thickening as depth increases.

On the other hand, the calibrated conductivity field could not detect the smaller-scale detailed layering of the soil structure. As a result, the simulated temperature field can only reproduce the dynamics of the average temperature signals, without the small temporal scale fluctuations observed in the DTS data. This may also be imputed to the selected inversion approach, which relies on Kriging, that typically results in a smoothing of the calibrated parameters.

The temperature time series collected in the experiment presented a superposition of fluctuations that could hardly be differentiated from the actual groundwater temperature dynamics. For instance, they present daily oscillations, presumably correlated to the effect of atmospheric temperature fluctuations on the interrogator electronics, that may conveniently require continuous dynamic calibration baths^45^ for the DTS interrogator, not employed here. Moreover, they may also carry the signature of in-well temperature phenomena, such as responses to pumping-induced fluctuations of the water column, which the model cannot account for. These issues in the DTS data complicate the evaluation of the goodness of fit between simulated and observed temperatures.

Another potential limitation of the model for heat transport in porous media employed here is that it relies on the local thermal equilibrium assumption^39,46^, whose validity was not verified in the experimental framework. Considering that thermal equilibrium is delayed for large grain size sediments^46^ and the gravelly composition of the Settolo aquifer, we cannot exclude the possibility that the local thermal equilibrium hypothesis may be violated. Furthermore, the role of thermal dispersivity and its mathematical representation have not yet been well established in the literature, especially in heterogeneous porous media^11,47^. Finally, the mathematical model adopted by FEFLOW for the thermal dispersivity coefficient is linear with respect to the thermal front velocity^39,46^, while an alternative nonlinear theory has recently been proposed by Rau et al.^47^. Further research is needed to clarify the impacts of these uncertainties on heat transport modeling in alluvial aquifers.

To expand our knowledge on the complex hydrogeological features of the Settolo area, a downhole FO-DTS monitoring network could be installed at the entire aquifer scale for long-term passive temperature monitoring. In addition, performing active thermal tracer tests over shorter spatiotemporal scales could provide more insights on the convoluted system of paleo-channels characterizing the subsurface at the site. Indeed, active DTS could significantly mitigate the uncertainties in the boundary conditions and provide a more marked temperature signature, potentially improving both the data interpretation and the inversion of a numerical model. This is because the ambient temperature field dynamics would become of secondary relevance with respect to the artificial perturbations induced by the tests. Assessing groundwater-stream exchanges, Simon et al.^43^ found that while long-term passive DTS was able to detect and locate groundwater inflows over a long distance, it required assumptions about thermal properties and boundary conditions that induced high uncertainties on fluxes quantification, for which active DTS over short spatiotemporal scale proved superior. The authors remarked on the importance of the complementarity of the two approaches, which has the potential to provide an imaging of the spatial variability of groundwater inflows. At any rate, the monitoring system will greatly benefit from continuous dynamic calibration baths in future studies.

## Conclusions

Naturally occurring and pumping-induced groundwater temperature variations in a shallow heterogeneous aquifer were monitored in a group of piezometers using downhole FO-DTS in passive mode. The strong hydraulic connection between the aquifer and the Piave River is responsible for seasonal temperature fluctuations, superposed to sub-seasonal signatures mainly related to the pumping activity and the dynamics of the Funer Creek. Over a month-duration experiment, these short-term fluctuations proved sufficient to detect differential temperature anomalies even without active groundwater heating or warm water injection.

In the local-scale study area, where the DTS survey was conducted, the data revealed a complex temperature field, showing borehole-dependent and layer-dependent features that we could qualitatively relate to the spatial structure of the hydraulic conductivity. The proximity of the wells also made it possible to observe between-well contiguities and dissimilarities in the response to the external forcings, providing a three-dimensional perspective to our understanding of the processes. Specifically, we were able to evaluate the likelihood of boreholes being located in or outside highly conductive paleo-channels characterizing the aquifer and to identify some layers that quickly respond to the nearby pumping activity.

In our field application, the absence of continuous calibration baths for the DTS system, possibly combined with often non-sharp temperature fluctuations, introduced some noise and bias in the data, which were only partially corrected thanks to the presence of a monitoring network that served as control. Highly complex hydrogeological structures, uncertain boundary conditions, noisy data to be used in the calibration objective function, and the high computational cost of the 3D model hampered the numerical inversion of the hydraulic conductivity. Moreover, the selected calibration approach may be inadequate to detect heterogeneities at such a level of detail.

The resulting conductivity field confirmed the main coarse-scale features but could not capture the smaller-scale detailed layering of the soil properties that emerged from the data analysis. However, the model resulting from the inversion could reproduce the overall temperature field, confirming strong heterogeneities at the site, even though it could not reproduce the observed small-scale temperature oscillations.

At this stage, the information provided by the observed DTS temperatures unraveled unique qualitative features of the subsurface structure, whose quantitative counterpart would require a much more controlled experimental setup to be determined with high accuracy. Further investigations, such as active thermal tracer tests, should be conducted to support and complement the results. We conclude that, in alluvial aquifers where seasonal or sub-seasonal temperature variations occur, passive FO-DTS is a valuable tool that can provide insight into the spatial variability of the hydraulic conductivity field at high resolution, potentially even at large spatiotemporal scales.

### Supplementary Information

Supplementary Information.

## Data Availability

The datasets generated during and/or analysed during the current study are available in the online repository: http://doi.org/10.25430/researchdata.cab.unipd.it.00001244.

## Code availability

The codes used to analyze the datasets of the current study are available in the online repository: http://doi.org/10.25430/researchdata.cab.unipd.it.00001244.
