# Supplementary material for: Fiber optics passive monitoring of groundwater temperature reveals three-dimensional structures in heterogeneous aquifers
Source: Sci Rep. 2024 Apr 10;14:8430. doi: 10.1038/s41598-024-58954-3 (PMC11006848; doi:10.1038/s41598-024-58954-3)
Supplement: Supplementary file 1 — Supplementary Information. [file 41598_2024_58954_MOESM1_ESM.pdf]

Supplementary Information of the research paper

Fiber optics passive monitoring of groundwater temperature reveals three-dimensional structures in heterogeneous aquifers

Davide Furlanetto[1], Matteo Camporese[1,*], Luca Schenato[2,3], Leonardo Costa[1], and Paolo Salandin[1]

[1]University of Padova, Department of Civil, Environmental, and Architectural Engineering, Padova, 35121, Italy
[2]National Research Council, Research Institute for Geo-Hydrological Protection, Padova, 35127, Italy
[3]now University of Padova, Department of Information Engineering, Padova, 35131, Italy
[*]matteo.camporese@unipd.it

Temperature profiles in the subsurface

To exemplify how temperature profiles in the presence of lateral flow can potentially be used to infer information about the spatial distribution of the subsurface properties, we report the results of a simple numerical experiment. Two scenarios are considered: one in which the aquifer is homogeneous and one in which the aquifer presents layers of lower and higher hydraulic conductivity, as schematized on the right of Figure S1. The temperature profiles after 30 simulation days at the midline of the domain are shown in the plot on the left of Figure S1. Compared to the homogeneous case, the temperature profile in the layered aquifer displays characteristic deviations that are indicative of different arrival times of the thermal front. In the more conductive layer, where advective heat propagation is more significant due to higher flow rates, heat propagates more rapidly, and the temperature reaches higher values in advance with respect to the other layers. Conversely, in the layer where the hydraulic conductivity is lower, heat propagation is delayed by the lower flow, resulting in a temperature that is not much different from the initial condition. If the inflowing water were colder with respect to the resident water, the deviations of the profile would have been of the opposite sign. Naturally, to observe this type of deviations in the profile, lateral inflowing water should have a significantly different temperature with respect to the resident water. In situations where this does not occur, active thermal tracer tests can be conducted to induce artificial temperature perturbations.

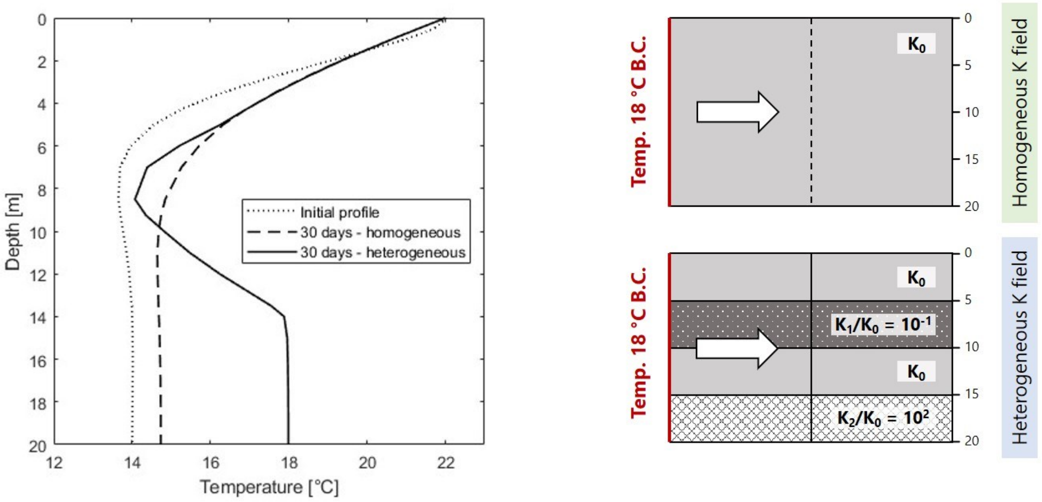

Figure S1 A simple simulation showing the influence of lateral flow on temperature profiles. On the right, a schematic of the two considered scenarios. On the left, temperature profiles obtained after 30 simulation days. A lateral hydraulic gradient of 1.7% was employed in the simulations.

Description of the full-scale 3D model

A full-scale 3D finite element model of the entire Settolo aquifer domain was implemented using FEFLOW 7.5 (DHI) to retrieve adequate boundary conditions for the local-scale model. The full-scale model boundaries are depicted by the black line in Figure 1b of the manuscript. Hydraulic heads and temperatures measured by point sensors in N12, PiaveUP, and p07 were employed as Dirichlet boundary conditions. The average pumping rates measured in BASE, CA, and SG were set as sink terms. The difference between daily precipitation and reference evapotranspiration (computed using the FAO Penmann-Monteith formula) was imposed on the surface together with the daily average air temperature.

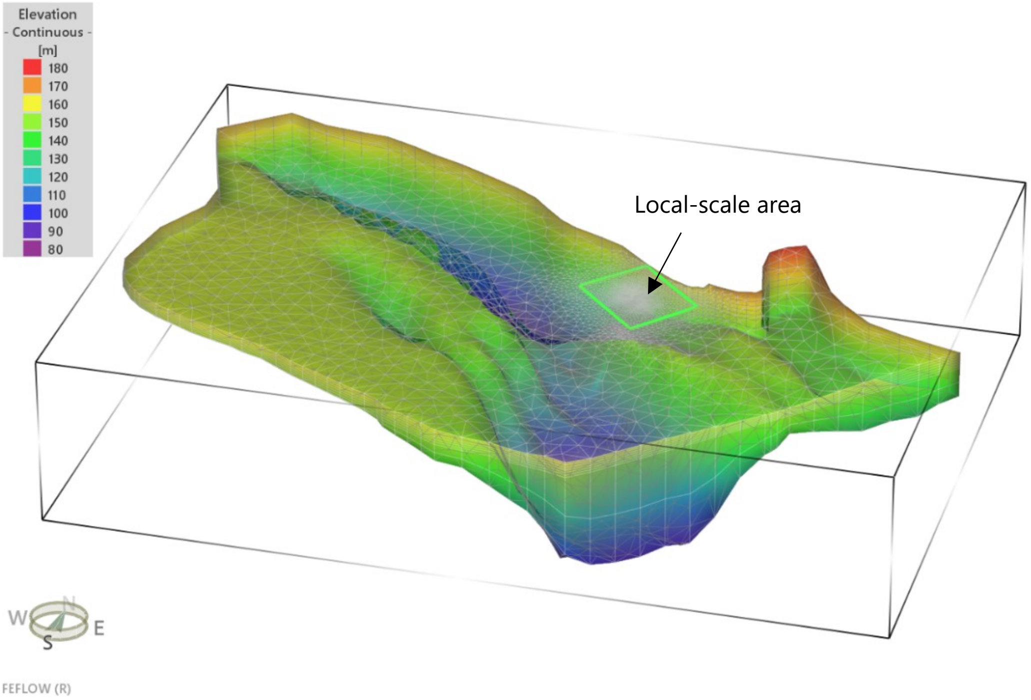

Figure S2 Mesh of the 3D full-scale Settolo aquifer model with calibrated bottom elevation. The color scale represents the elevation above the mean sea level. The green line delineates the position of the local scale area.

Preliminary data analysis and early modeling results showed that the thermal front originated from the Piave River, the main driving forcing of the temperature dynamics at the site, takes 7-9 months to reach the local-scale experimental area. Considering also the influence of the initial conditions on the model, a period of at least one year ahead of the experiment period (September 2020 – October 2020) was required to produce reliable boundary conditions for the local-scale model. Unfortunately, water level and temperature measurements necessary to impose the boundary conditions for the full-scale model were not available after 2018.
Therefore, the following approach was adopted: (1) selection of a model period with adequate data availability; (2) calibration of the bottom (Figure S2), of the hydraulic conductivity and the thermal dispersivity fields (Figure S3) in that period; (3) reconstruction of the boundary conditions in the DTS experiment period for the full-scale model based on other environmental data; (4) forward simulation combining reconstructed boundary conditions and calibrated parameter fields to retrieve the local-scale area boundary conditions. The simulated hydraulic head in some observation wells is compared to the measured ones in Figure S4. The simulated and observed temperatures in p03 are shown in Figure S5.

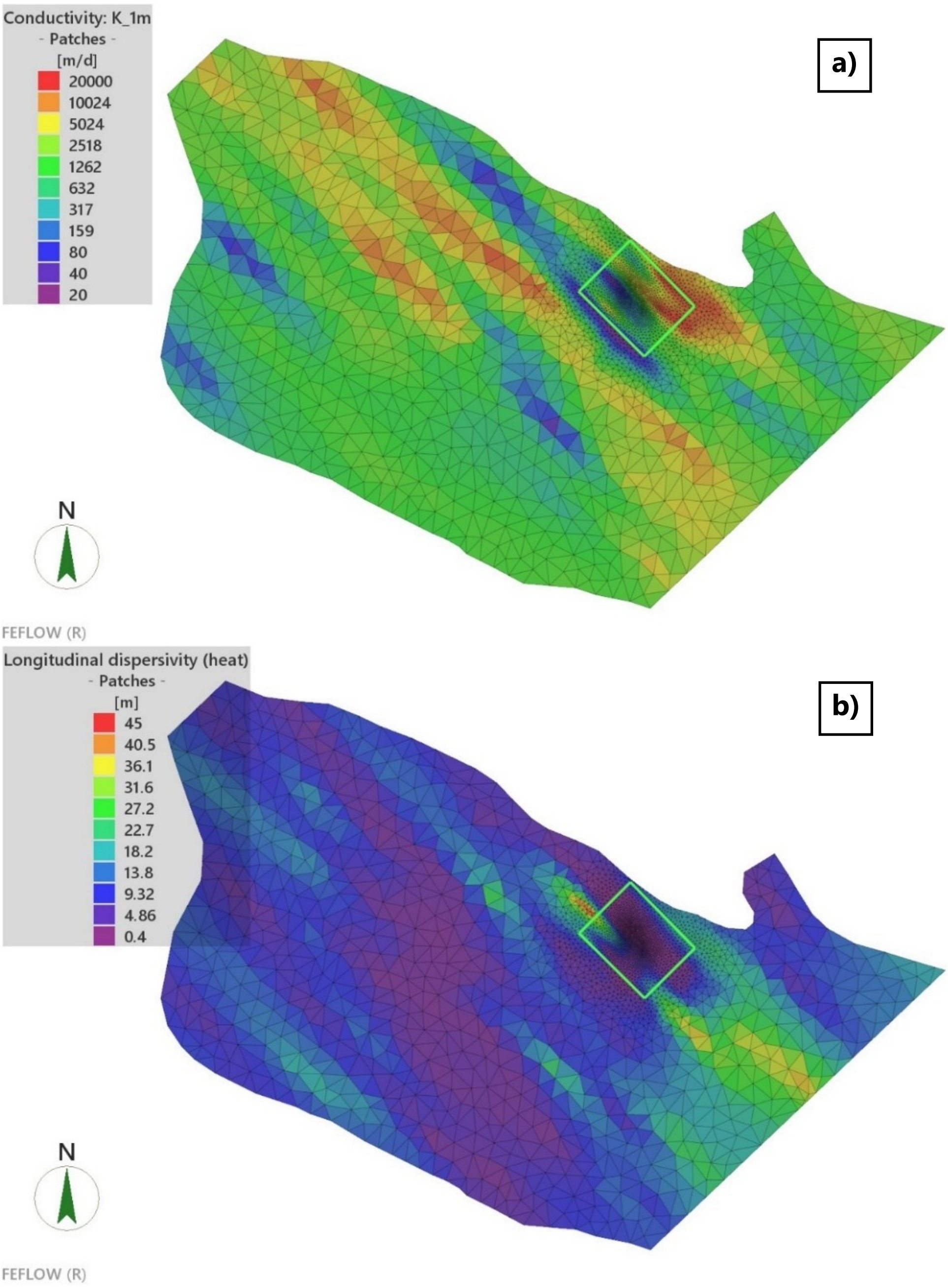

Figure S3 *(*a) Calibrated horizontal hydraulic conductivity field and (b) calibrated longitudinal thermal dispersivity field.

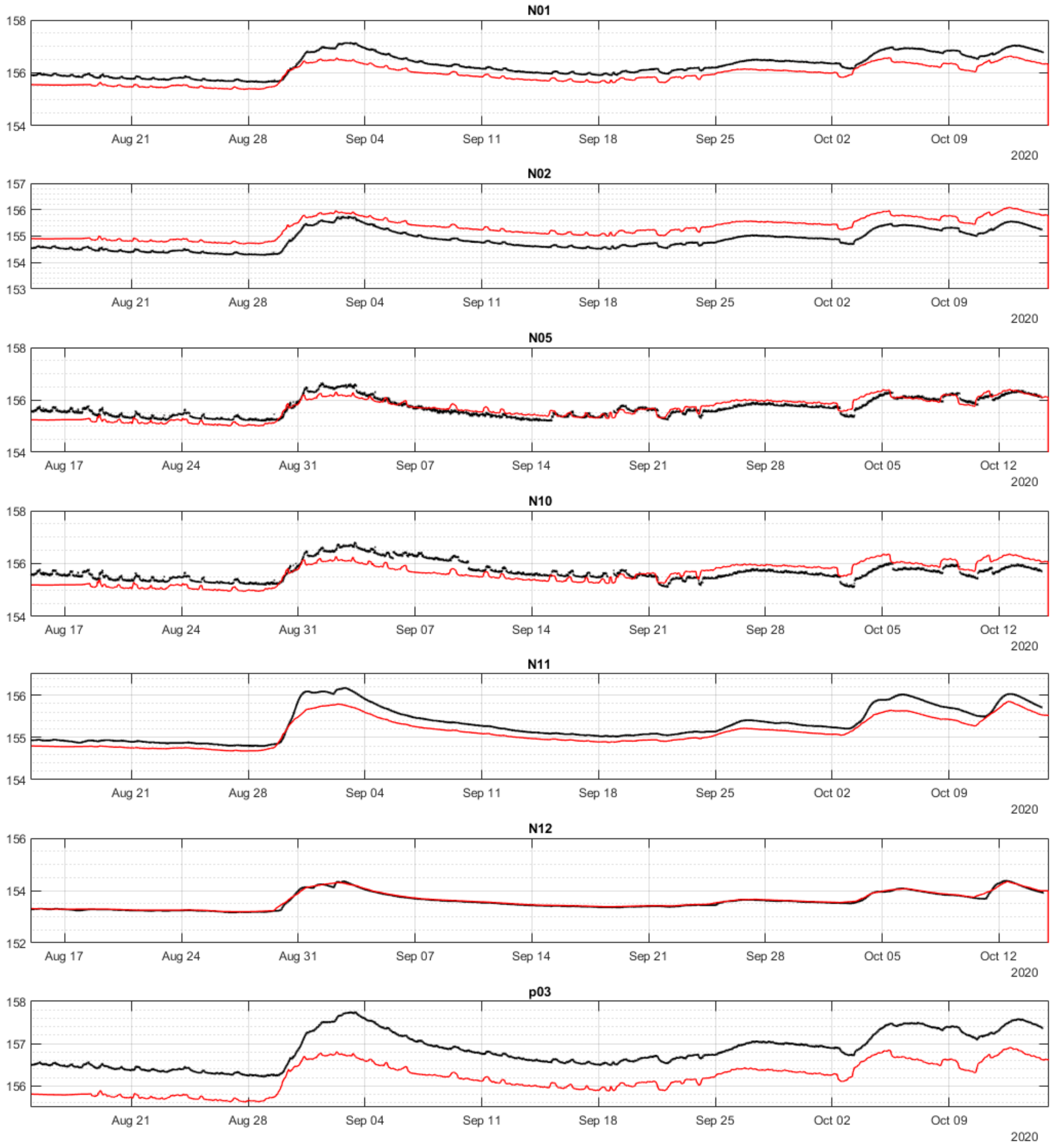

Figure S4 Full-scale 3D model simulated (red) and observed (black) hydraulic head (m), after calibration, at the observation wells.

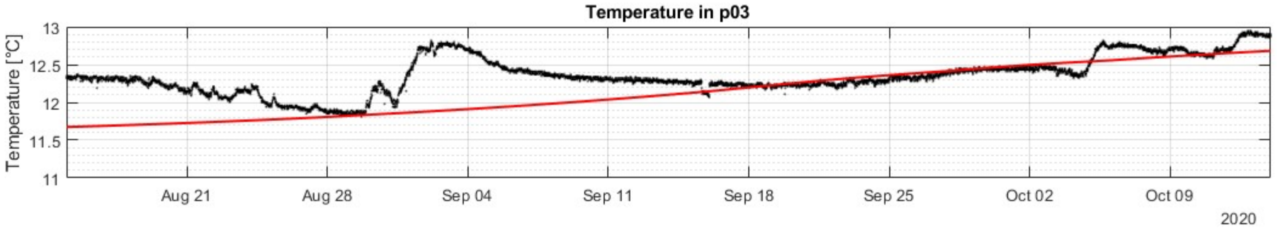

Figure S5 Full-scale 3D model simulated (red) and observed (black) point temperature (°C) in well p03.

The daily average air temperature was used to reconstruct the Piave River water temperature, whose data are completely missing after April 2018 due to the loss of the probe indicated as “PiaveUp” in Figure 2. The upper plot of Figure S6 shows the daily average air temperature recorded at the meteorological station of Bigolino, located about 3 km South-East from the experimental site. The lower plot of Figure S6 shows the daily average water temperature of the Piave River as recorded by the probe “PiaveUp” when available (black), and the water temperature reconstructed based on the air temperature data (red) to fill the missing observations.

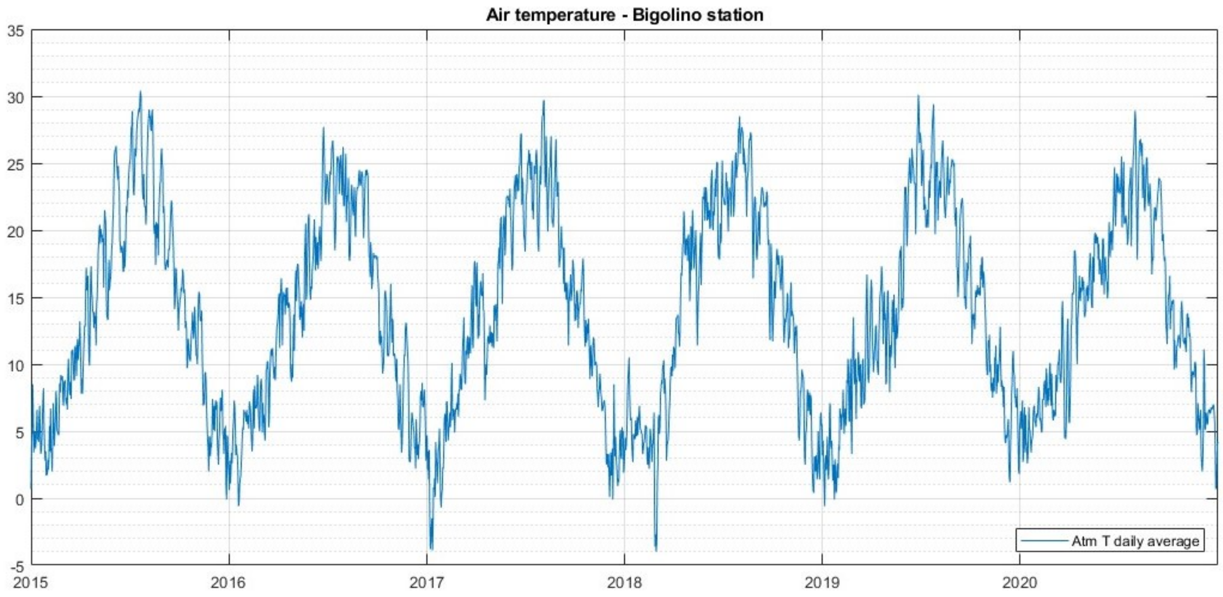

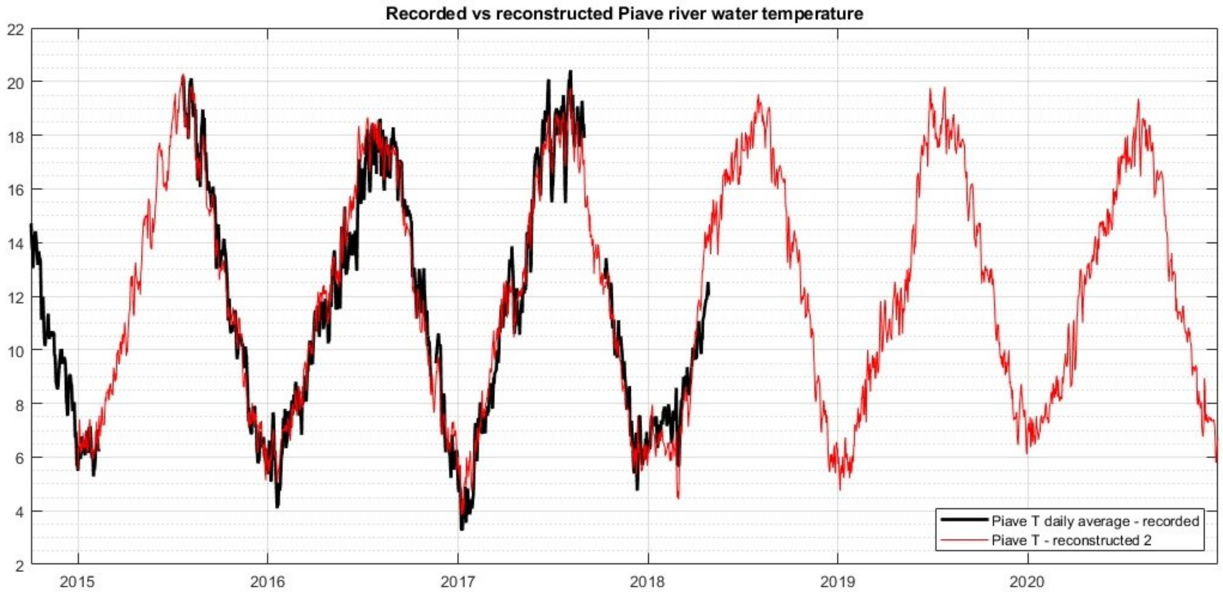

Figure S6 (Upper plot) Daily average air temperature (°C) at the station of Bigolino, located about 3 km South-East from the experimental site. (Lower plot) Daily average water temperature (°C) recorded by the “Piave_up” probe (black) and reconstructed based on air temperature data (red).

Preliminary data analysis

A detailed map with the positioning of the cable is shown in Figure S7.

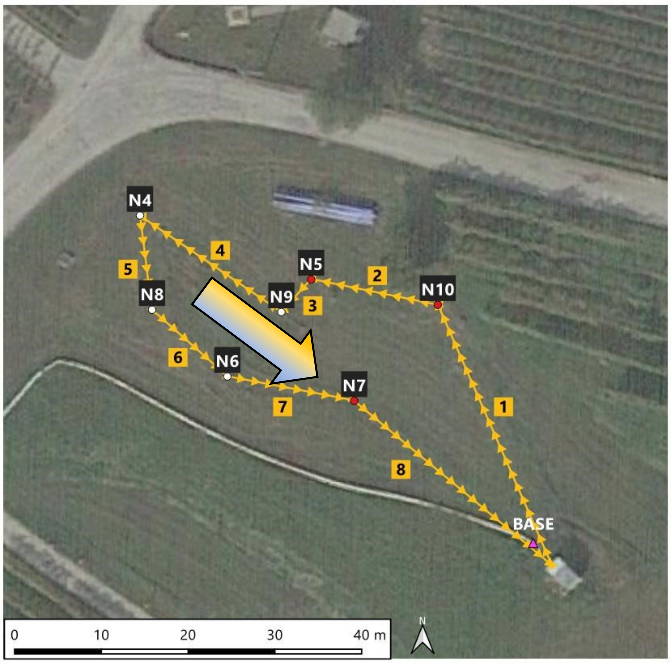

Figure S7 Detailed positioning of the fiber optic cable with numbering of the cable segments connecting different boreholes. The direction of the large arrow indicates the mean flow direction, while the filling color represents the groundwater temperature gradient (warmer towards the North-East, colder towards the South-West).

The space- and time-averaged temperature signals collected by the sections of the fiber-optic cable inserted into each borehole are displayed on the left of Figure S8. The space- and time-averaged temperature signals collected by the fiber optics cable sections connecting different boreholes (as numbered in Figure S7), are displayed on the right of Figure S8.

Considering that the mean groundwater flow direction is approximately from North-West to South-East (as shown by the direction of the large arrow in Figure S7 and Figure 2c), the analysis of these average temperatures reveals a transversal temperature gradient with respect to the mean flow direction, with average groundwater temperature decreasing from North-East to South-West, as represented by the colors of the arrow in Figure S7. The same trend cannot be observed in the sections of cable connecting different boreholes (Figure S8, right), which are laid on the ground surface, thereby excluding an artifact of the interrogation system on the progressive of the cable.

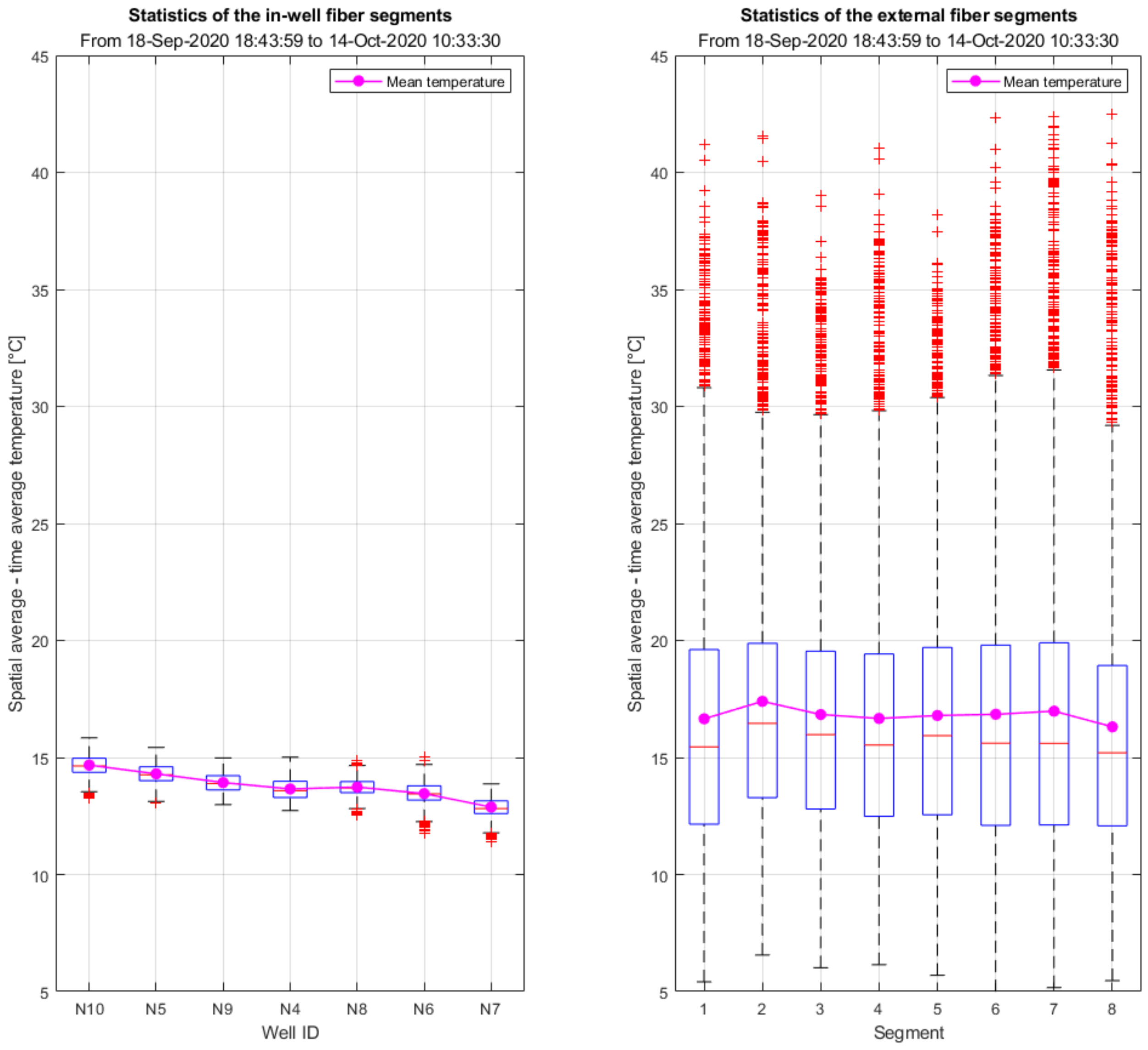

Figure S8 Space- and time-averaged temperature signals collected by the fiber optic cable sections within the boreholes (left) and connecting different boreholes (right).

Time series comparison

Figure S9 shows the raw temperature time series recorded in N05 and N10 at the same location of the point probe. The same signals are also shown after applying a moving average of 5.5 hours and an offset bias correction of -0.78°C (DTS corrected). The temperature time series recorded by the point sensors in N05 and N10 is also shown.

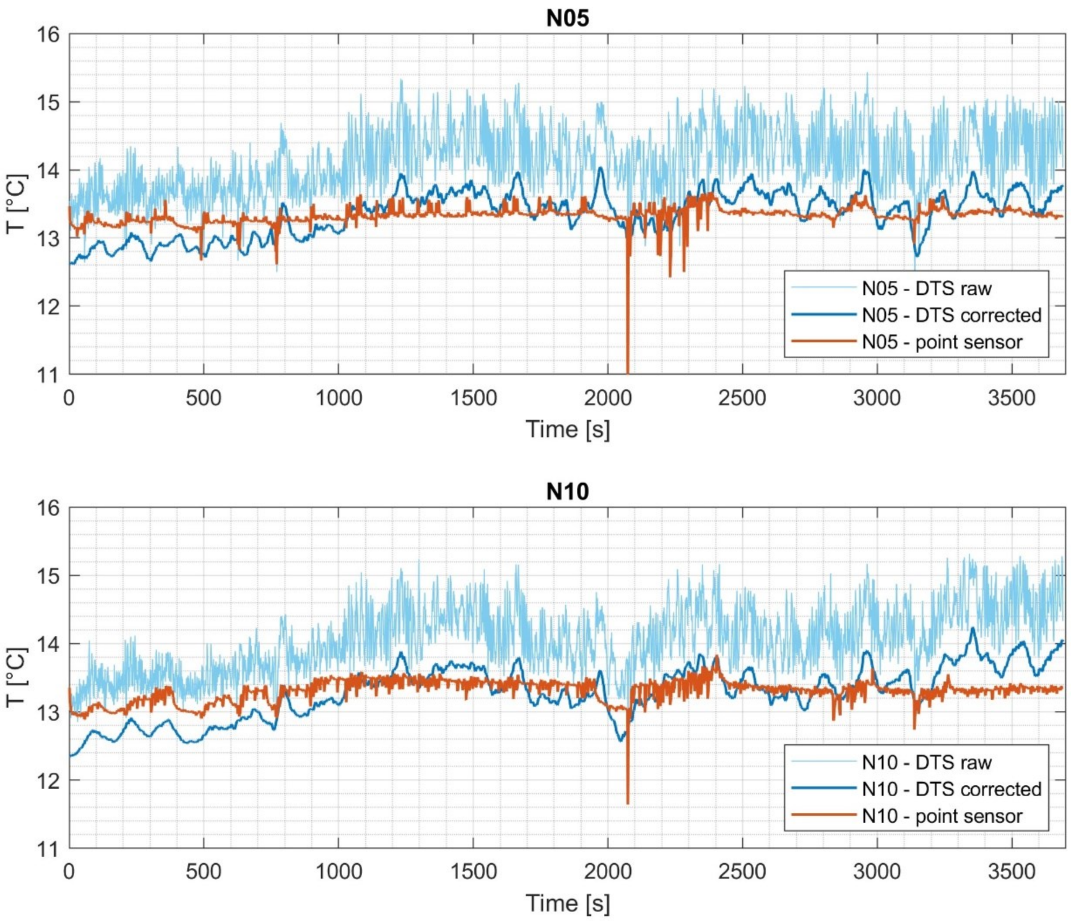

Figure S9 Comparison of the temperature time series recorded by the fiber-optics, before and after the application of the correction with the temperature time series recorded by the point probes in N05 and N10.

Correlation matrix and cluster analysis

A complete Pearson correlation matrix of the FO-DTS temperature time series recorded at different depths in the investigated piezometers between September 18th, 2020, and October 14th, 2020, is shown in Figure S10.

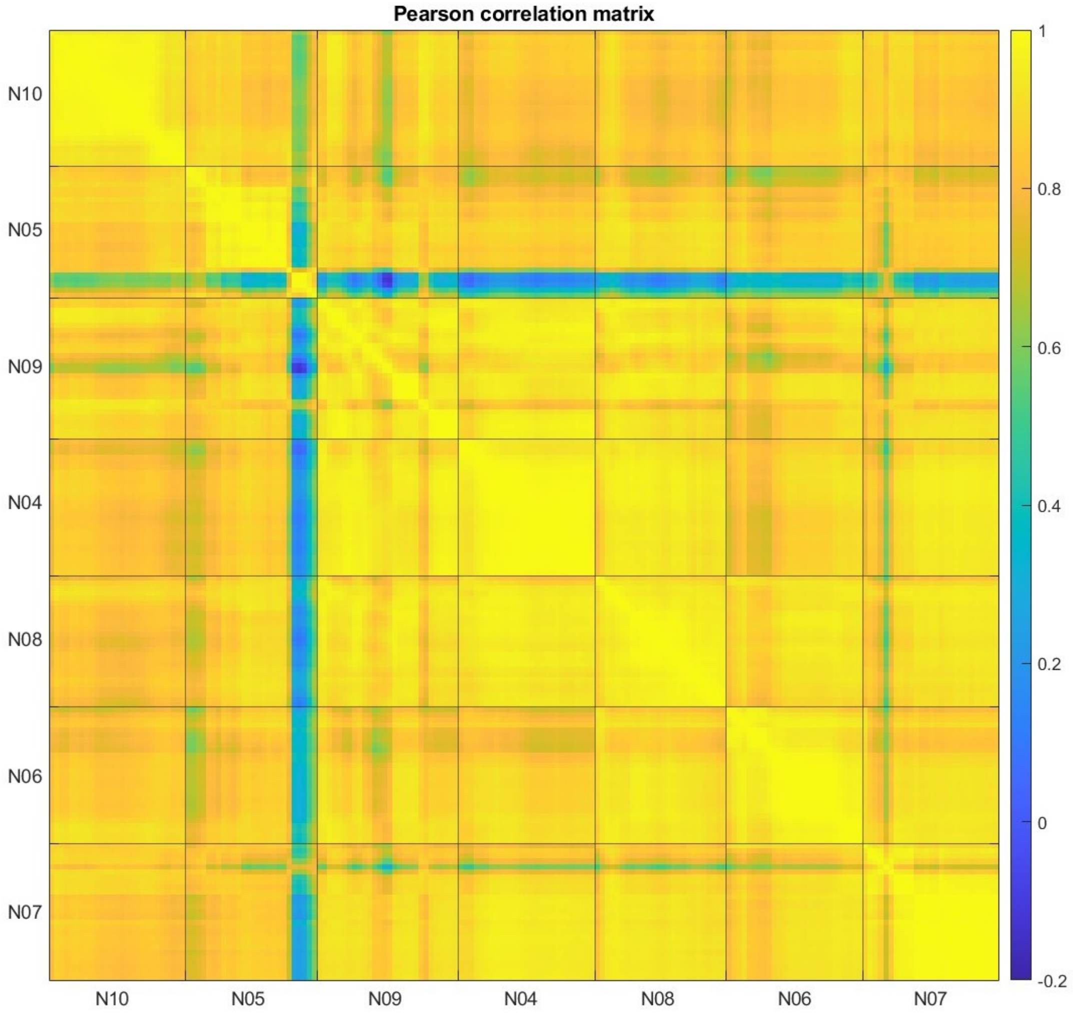

Figure S10 Pearson correlation matrix between temperature time series in all the investigated boreholes at depths larger than 5 m.

The dendrograms resulting from a cluster analysis of the DTS time series in the four representative piezometers (N10, N05, N09, N07) at different depths are shown in Figure S11.

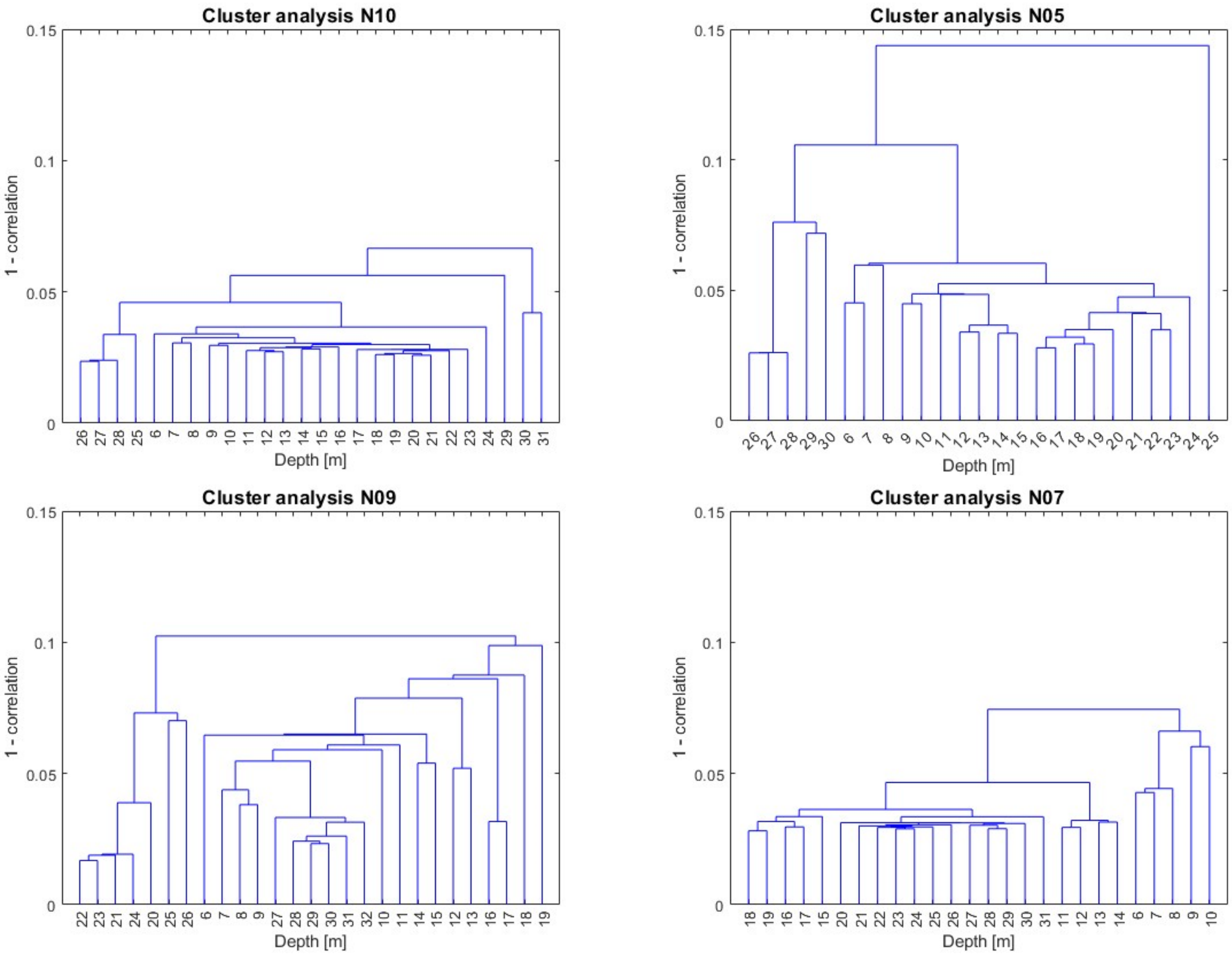

Figure S11 Dendrogram plots of the cluster analysis of temperature time series at depths larger than 5 m in the four representative boreholes.

Results of the simplified 1D heat transport model

The temperatures simulated by the simplified 1D heat model in the four representative boreholes, as a function of time and depth, are shown in Figure S12.

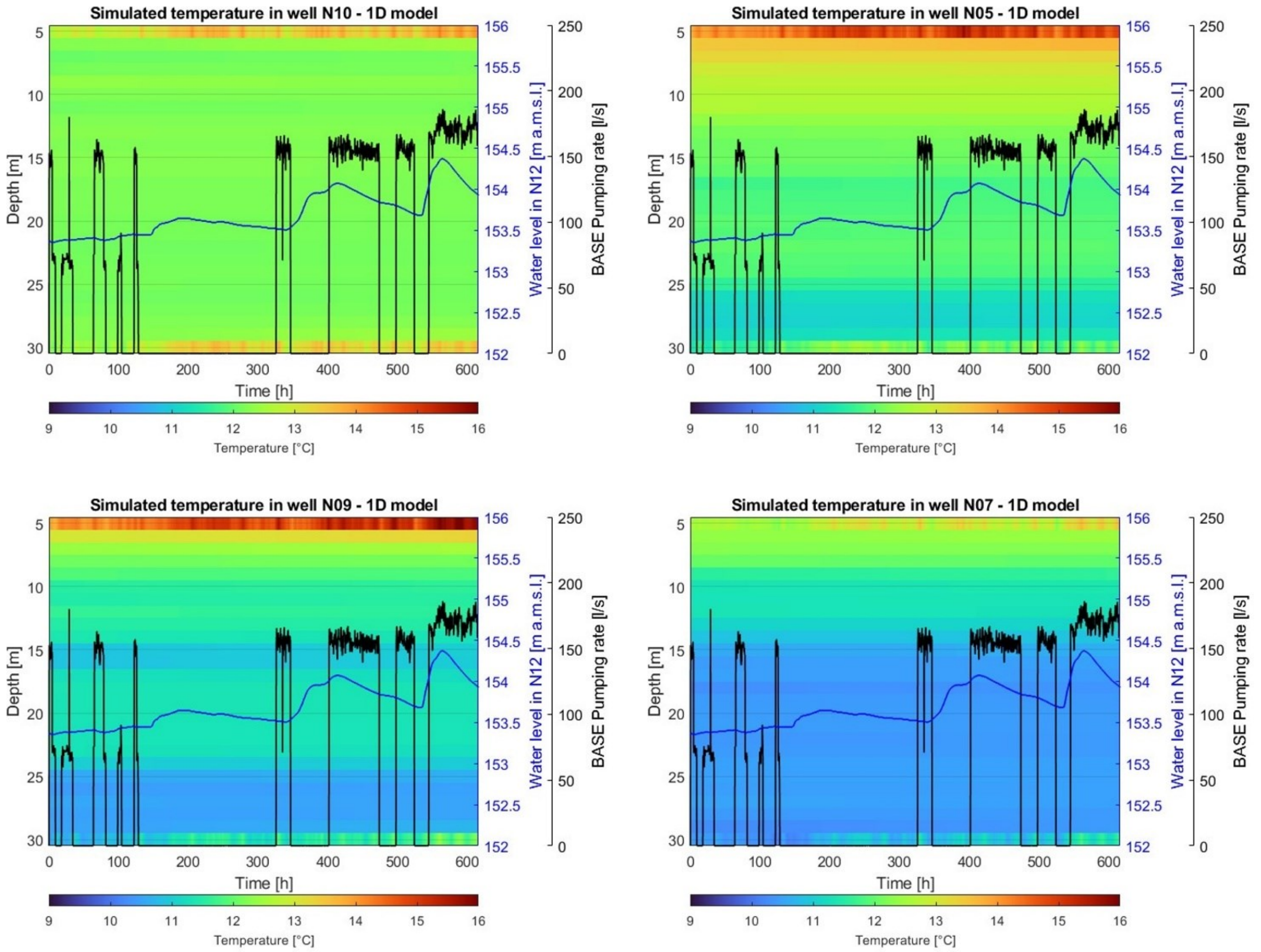

Figure S12 Temperatures simulated by the 1D heat model as a function of time and depth in boreholes N10, N05, N09, and N07. Time is indicated in hours from the beginning of the experiment. Color bars indicate the temperature in °C. Water level measured in borehole N12 in m above the mean sea level, along with BASE well pumping rate in l/s, are also reported. The water level in N12 is shown as it is representative of the piezometric surface dynamics, mainly controlled by the Piave River and undisturbed by the pumping process.

The 1D model simulates the evolution of the temperature profile, starting from an initial condition based on the measurements, under the purely hypothetical assumption of no lateral heat fluxes. The boundary condition imposed at the top of the column consists of the temperature time series collected by the cable section just below the water table (but still submerged), which is likely influenced by the air temperature variations. The temperature profile is markedly different for the four boreholes because of the different initial conditions. The results of this simplified 1D model suggest that the vertical heat propagation of air (and hence water surface) temperature variations is negligible, due to the large heat capacity of water; therefore, the actual temperature profile measured by the DTS can mainly be ascribed to lateral heat fluxes not accounted for in the model.
